# Retraction: CDX2/mir-145-5p/SENP1 Pathways Affect LNCaP Cells Invasion and Migration

**DOI:** 10.3389/fonc.2021.744978

**Published:** 2021-08-18

**Authors:** Frontiers Editorial Office

**Affiliations:** Frontiers Media SA, Lausanne, Switzerland

The Journal retracts the 12 June 2019 article cited above for the following reasons:

Following publication of the original article, concerns were raised regarding the integrity of the images in the published figures. During the investigation, the authors submitted a correction article which was published during our investigation and which, upon further scrutiny, did not resolve the fundamental issues of image integrity. In the end, the authors have failed to provide a satisfactory explanation during the investigation, which was conducted in accordance with Frontiers’ policies.

This retraction was approved by the Chief Editors of Frontiers in Oncology and Chief Executive Editor. The authors did not agree to this retraction.

